# Enhancing bioactivity of 3D-printed porous scaffolds with self-assembling peptide hydrogels for cartilage tissue engineering

**DOI:** 10.1186/s41205-026-00330-0

**Published:** 2026-06-24

**Authors:** Michael Kainz, Damien Djian, Sharanya Sankar, Kerimcan Bagci, Shabnam Hemmati-Sadeghi, Isabel Caetano da Silva, Michael Sittinger, Elena Guillén, Tilo Dehne

**Affiliations:** 1 Functional Surfaces and Nanostructures, Profactor GmbH, Steyr-Gleink, 4407 Austria; 2 Elkem Silicones France SAS, Saint Fons, 69190 France; 3 3-D Matrix Europe SAS, Caluire-et-Cuire, 69300 France; 4 Laboratory for Tissue Engineering, Department of Rheumatology and Clinical Immunology, Charité – Universitätsmedizin Berlin, 10117 Berlin, Germany

**Keywords:** ECM mimicry, Hybrid scaffold, Hydrogel augmentation

## Abstract

**Background:**

Cartilage repair is challenging due to the tissue’s limited regenerative capacity. Synthetic 3D-printed scaffolds provide essential structural support, but typically lack the bioactivity needed for cell integration. A promising approach combines 3D-printed porous scaffolds filled with self-assembling peptide hydrogels, which serve as nanofiber scaffolds inside the macropores of the structural scaffold, creating a hybrid structure.

**Methods:**

The selection strategy for the 3D printing of the synthetic scaffolds was driven by two distinct cross-linking processes: a vinyl-ester based thiol-ene photopolymer crosslinked via free radical polymerization and printed with digital light processing, resulting in a stiff mechanical network and polydimethylsiloxane, namely AMSil™ 20503-50 from the AMSil™ 20,503 series, printed via liquid deposition modeling and crosslinked through polyaddition, which yields flexible scaffolds capable of adapting to dynamic mechanical environments. These properties make them suitable for load-bearing applications where structural integrity is paramount. Both 3D-printed scaffold types, characterized by interconnected macropores ranging from 0.8 to 1.2 mm, were augmented with a peptide hydrogel scaffold, such as RADA16 and IEIK13, that self-assembles inside the macropores to create a nanofiber network mimicking the extracellular matrix and enhancing bioactivity.

**Results:**

The hybrid structure, combining the macropores of a structural 3D-printed scaffold and the nanofiber network of the peptide hydrogel scaffold improved cell adhesion, proliferation, and differentiation. Comparative analysis showed that, while both RADA16 and IEIK13 hydrogels enhanced cell integration within the macropores, RADA16 was especially effective in supporting cartilage-like ECM formation.

**Conclusions:**

The creation of a hybrid scaffold with hierarchical porosity—integrating the structural macropores of a synthetic 3D-printed scaffold with the bioactive nanofiber network of a peptide hydrogel—addresses the limitations of purely structural scaffolds. The hybrid approach not only enhances fast and accessible scaffold fabrication but also accelerates the development of functional scaffolds.

## Background

The rapid advancement of 3D printing technologies has significantly contributed to the development of scaffolds for cartilage repair [1]. Synthetic materials are particularly attractive due to their 3D printing capabilities and mechanical properties [2]. However, their intrinsic lack of bioactivity poses a challenge, as these materials often fall short in supporting essential cellular processes such as attachment, proliferation, and differentiation, which are critical for tissue regeneration [3]. This shortfall arises because the mechanical properties alone are insufficient to support full tissue integration [4]. This deficiency limits their applications where bioactive interactions are crucial, such as in the regeneration of cartilage tissue.

Hydrogels are versatile biomaterials that provide an environment for cell colonization but typically lack the mechanical properties required for load-bearing applications [5]. By embedding hydrogels strategically within a stiffer matrix, such as a synthetic polymer scaffold, hybrid constructs can be created that combine the desirable attributes of both materials. This strategy has been particularly applied in bone tissue engineering using polymers such as poly(ε-caprolactone) (PCL), poly(lactic acid) (PLA), and poly(lactic-co-glycolic acid) (PLGA). One example is the integration of 3D-printed PCL scaffolds with hydrogels like alginate and gelatin, which, when combined with human mesenchymal stem cells, enhance scaffold bioactivity [6]. Similarly, the addition of injectable, thermo-sensitive chitosan hydrogels to 3D-printed PCL scaffolds improves cell seeding and osteoinductivity, as demonstrated by encapsulating rabbit bone marrow stem cells and bone morphogenetic protein (BMP)-2 within the chitosan matrix [7].

PLA microstructures, produced through fused deposition modeling, further illustrate the potential of hybrid constructs. When paired with photopolymerizable gelatin hydrogels containing RGD-conjugated gold nanoparticles and human adipose-derived stem cells, these structures promote osteogenic differentiation [8]. Building on these developments, PLA cages filled with a gelatin and alginate-based biogel carrier for BMP-2 have shown effective release and substantial bone regeneration in both in vitro and in vivo studies [9]. Additionally, 3D-printed PLGA scaffolds loaded with BMP-9 and P-15 peptide hydrogels have demonstrated significant improvements in repairing bone defects in rabbit models, enhancing both biological and mechanical properties [10].

In orthopaedic applications, 3D-printed porous titanium scaffolds fabricated via electron beam melting and filled with a simvastatin/poloxamer 407 hydrogel enhance neovascularization and osseointegration [11]. Additionally, titanium scaffolds loaded with antibacterial agents serve dual purposes in maxillofacial surgery, offering structural support while preventing infections [12]. Furthermore, hybrid constructs featuring heparin-functionalized collagen gels integrated with 3D-printed ceramic scaffolds have been developed to mimic the bone extracellular matrix (ECM), improving mesenchymal stem cell seeding efficiency and osteogenic differentiation through immobilized BMP-2 [13].

Beyond bone tissue engineering, hybrid scaffolds also hold significant promise in cartilage and osteochondral tissue engineering [14]. Notably, digital light processing (DLP) 3D-printed polyethylene (PEG) diacrylate based scaffolds filled with mesenchymal stem cells and soft biomimetic PEG-based hydrogels formulated with ECM analogs and tethered growth factors enhance cellular regeneration [15]. Stereolithography-based 3D-printed scaffolds using non-degradable commercial resins combined with injectable PEG-based photopolymerizable hydrogels encapsulated with bovine chondrocytes have shown potential in treating focal chondral defects by promoting neotissue growth [16]. Moreover, bioactive artificial auricular cartilage constructs, developed by integrating chondrocyte-laden gelatin methacrylate with PLA scaffolds, demonstrate favorable biomechanical properties and support chondrocyte viability, highlighting promising applications in auricle regeneration [17].

The reviewed literature typically incorporates growth factors or cells into amorphous hydrogels. In contrast, our study avoids using these bioactive agents, choosing instead peptide-based hydrogels that self-assemble into nanofiber networks. These networks promise to better mimic the ECM, enhancing mechanical support and cellular interactions compared to amorphous networks. This approach was selected with future regulatory certification in mind, aiming to maintain a hybrid scaffold as a medical product rather than advancing it to a medicinal drug or an advanced therapy medicinal product.

We investigate the integration of two peptide hydrogels, differing in amino acid composition and nanofiber network, into 3D-printed synthetic scaffolds. RADA16, a widely used self-assembling peptide hydrogel, has shown efficacy in promoting in vivo bone regeneration [18]. Although RADA16 has mainly been studied in bone tissue engineering, its physicochemical properties also make it well suited for cartilage repair. The peptide self-assembles into β-sheet nanofibers that form a hydrated, ECM-like network resembling the collagen fibrillar structure of native cartilage. Previous studies have shown that self-assembling peptide hydrogels support chondrocyte viability and glycosaminoglycan synthesis [19] and enhances chondrogenic differentiation potential of BMSCs in vitro when loaded with transforming growth factor (TGF)- β1 [20]. Owing to its nanofibrillar architecture, injectability, and excellent biocompatibility, RADA16 was therefore selected as a representative self-assembling peptide hydrogel to evaluate its capacity to bioactivate synthetic 3D-printed scaffolds for cartilage regeneration.

IEIK13, supports the production of nasal cartilage matrix and has been utilized with BMP-2 + insulin + triiodothyronine to aid in cartilage repair [21]. The hydrogel has also been used as scaffold to support articular chondrocytes and the repair of articular cartilage defects, when implanted alone or with chondrocytes [22]. Our study aims to showcase their potential in a cell-free context by integrating them into 3D-printed scaffolds to support cellular colonization. This hybrid approach leverages the rapid fabrication capabilities of porous structures using 3D printing while embedding a nanofiber network within the scaffold pores. Specifically, our focus is on synthetic photopolymer- and polydimethylsiloxane (PDMS)-based materials due to their contrasting yet complementary properties for cartilage scaffolds. The photopolymer offers stiffness, conversely, PDMS, with its excellent elastomeric properties, offers flexibility and resilience suitable for load-bearing regions of cartilage. Despite their mechanical differences, both materials can be tailored via 3D printing for cartilage scaffolds. By combining these materials with peptide hydrogels, the 3D-printed scaffolds are augmented with a synthetic ECM, enhancing colonization properties.

This study demonstrates a fully cell- and growth-factor-free hybrid scaffold concept that integrates self-assembling peptide nanofiber hydrogels (RADA16, IEIK13) into both stiff photopolymer and flexible PDMS 3D-printed matrices, thereby bioactivating otherwise inert materials and enabling cartilage-like ECM formation through a scalable, ISO-compliant manufacturing workflow.

## Methods

### Materials

The study utilized two types of synthetic materials for 3D-printed scaffolds: a vinyl-ester based thiol-ene photopolymer for digital light processing [23] and a PDMS-based elastomer (AMSil™ 20503-50 from the AMSil™ 20503 series, Elkem Silicones, Saint Fons) for liquid deposition modeling. The AMSil™ 20,503 series elastomers are two-component silicone elastomers that crosslink at high temperature by polyaddition reaction. Additionally, two synthetic peptide hydrogels were employed: RADA16 (1% slution, 3D-Matrix Europe SAS, Caluire-et Cuire) and IEIK13 (1.3% slution, 3D-Matrix Europe SAS, Caluire-et Cuire). The hydrogels used in this research are non-clinical, research-grade peptide formulations. They are distinct from commercially marketed RADA16-based medical devices (e.g., haemostatic products), which have specific approved indications and instructions for use. Nothing in this article should be interpreted as guidance for clinical application or off-label use of any marketed product.

### Fabrication of 3D-printed scaffolds

**Fig. 1:**
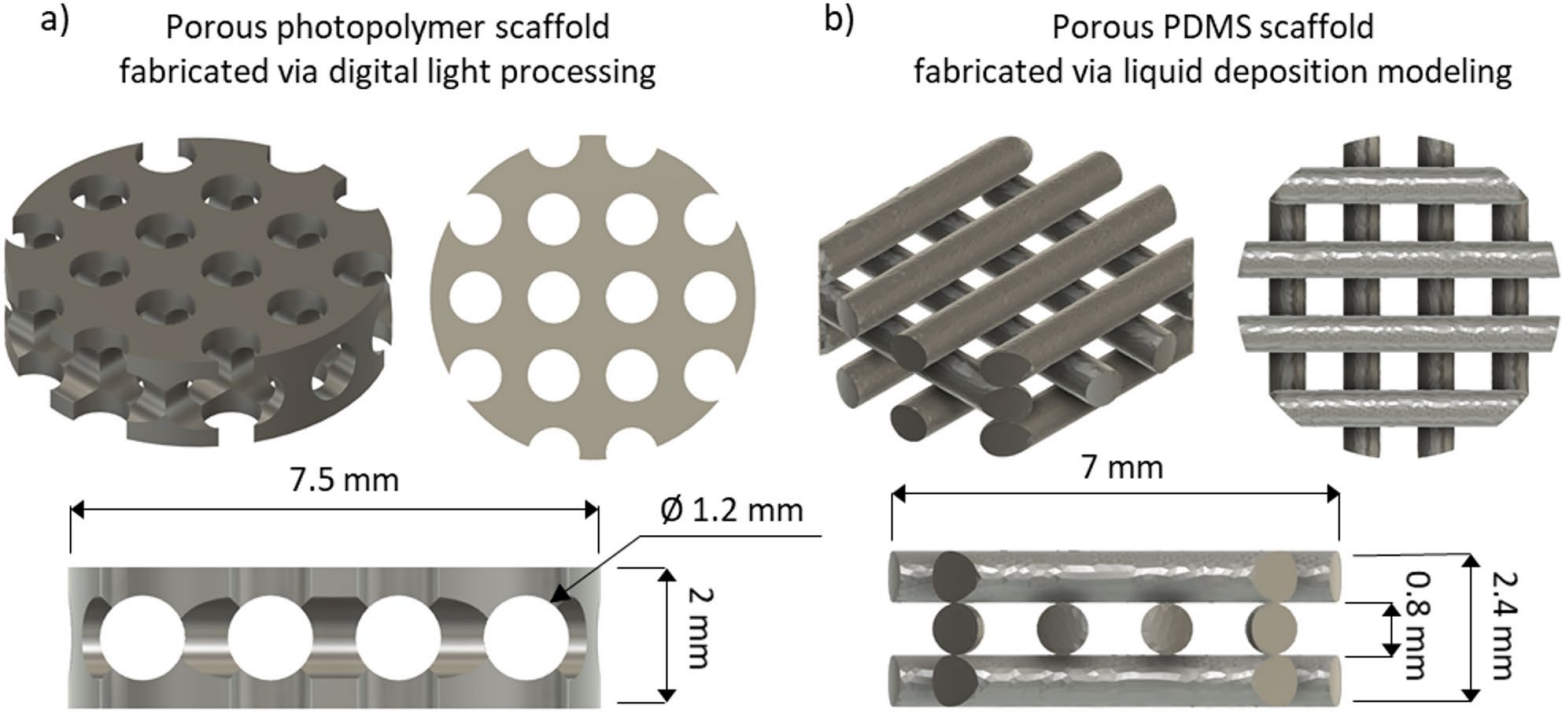
Design of 3D-printed (**a**) photopolymer and (**b**) PDMS scaffolds

For static culture, designs were used to meet the requirements for cartilage use cases (2.0 –2.4 mm height) and the maintenance with cells under static cell culture conditions (7 –7.5 mm diameter). Scaffold designs were tailored with a pore size ranging from 0.8 to 1.2 mm, Fig. 1. Photopolymer scaffolds were fabricated using DLP (Original Prusa SL1S SPEED, Prusa Research a.s., Czech Republic). Printing was conducted with a layer thickness of 25 μm, an initial exposure time of 15 s (3 faded layers), and subsequent layer exposure time of 2.8 s. After printing, the scaffolds were washed in ethanol (EtOH) in an ultrasonic bath for 10 min to remove any unreacted resin. PDMS scaffolds were printed via liquid deposition modeling (Delta Tower Fluid MT, Deltatower GmbH, Switzerland), utilizing a metallic nozzle tip of 437 μm and a layer height of 400 μm. After printing, the scaffolds were cured at 70 °C for 1 h and then at 150 °C for 3 h.

### Hybrid scaffold system: peptide hydrogel loading of 3D-printed scaffolds

In preparation of the biological evaluation, washing protocols were applied to remove potential unreacted components. Briefly, photopolymer scaffolds were washed 4 × 24 h at 37 °C in 75% EtOH followed by 4 × 24 h at 37 °C in cell culture medium under constant agitation. Photopolymer scaffolds were sterilized with 80% v/v EtOH for 1 min followed by > 2 h drying. PDMS scaffolds were washed as follows, 24 h immersion in 50% EtOH at 70 °C (this aims to provoke the swelling of the PDMS to wash out un-crosslinked residue), 12 h drying in closed ventilated area, 30 min drying at 150 °C. The drying steps aim to erase any footprint of water or EtOH in or on the material. PDMS scaffolds were sterilized by autoclaving.

The hydrogels RADA16 and IEIK13 were incorporated into 3D-printed scaffolds by immersion in an excess volume (> 500 µL) for 10 min in a 2 mL non-conical centrifugation tube (Eppendorf, Safe-Lock Tubes 2.0 mL). Potential air bubbles were removed by centrifugation at 2000 g. Gelation of the hydrogels was induced by transferring the gel-laden structure to 1 mL of a physiological solution (cell culture medium with 10% fetal bovine serum (FBS)).

### Cytocompatibility

Washed and disinfected 3D-printed scaffolds (photopolymer with 80% v/v EtOH for 1 min, > 2 h drying, PDMS by standard autoclavation) were subjected to a cytotoxicity test according to the ISO 10993-5 standard. The obtained extracts were tested on three cell types: MC3T3-E1 and C2C12 are standard cell lines with distinct properties for detecting toxic effects, and porcine chondrocytes, the most abundant cell type in knee cavity and meniscus.

Porcine chondrocytes were isolated from the femoral condyle of 4 sacrificed 6–8-month-old female pigs (100–125 kg) as described previously [24]. Tissue samples were obtained from a slaughterhouse, therefore, no study approval was necessary. Briefly, cartilage slices (2–3 mm) were incubated for 19 h at 37 °C in spinner flasks, including an enzymatic solution containing Roswell Park Memorial Institute Medium (RPMI) medium (Merck), 10% FS (Sigma), 100 U/mL penicillin, 100 µg/mL streptomycin, 333.3 U/mL collagenase II (Merck), 1 U/mL collagenase P (Roche, Mannheim, Germany) and 33.3 U/mL hyaluronidase (Sigma-Aldrich, Steinheim, Germany). Cells were centrifuged at 400 g for 15 min, resuspended in complete RPMI medium (RPMI with 10% FS, 100 U/mL penicillin, 100 µg/mL streptomycin) and plated at a density of 20,000 cells/cm^2^. The medium was changed after 72 h and at 90% cnfluence, cells were detached with 0.05% typsin/ethylenediaminetetraacetic acid (EDTA) and sub-cultured up to passage 3. The murine myoblast cell line C2C12 and calvaria pre-osteoblast cell line MC3T3-E1 (German Collection of microorganism and cell cultures, Braunschweig, Germany) were cultured in Dulbecco’s minimal essential medium (C2C12) or alpha minimal essential medium (MC3T3-E1) supplemented with 10% FS (Sigma), 20 mM HEPES-buffer (Sigma), 100 U/mL penicillin, 100 µg/mL streptomycin (Merck), and 2 mM l-alanyl-glutamine (Sigma).

Cell viability was assessed via metabolic activity measurement using the Cell Counting Kit-8 (CCK-8) (Roth). The working solution was applied 1:20 in cell culture medium and absorbance was measured at 450 nm (corrected by 650 nm) with a spectrophotometer (Synergy HT, Biotek instruments). Before preparing extracts from material specimens, 5,000 cells were seeded in 96-well plates. Extracts of scaffolds were generated according to ISO 10993-12:2021 by incubating 3D-printed specimens in 1 mL cell-specific medium at 37 °C for 24 h in a 24-well under continuous shaking.

Hydrogels (225 µl) were casted in custom-made PLA (engineering PLA, 3DK.Berlin Trading GmbH, Berlin, Germany) moulds (inner/outer diameter 12/14 mm, depth 1/3 mm) and gelified in phosphate-buffered saline (PBS) before extraction under the same conditions. Extracts and additional dilutions in fresh cell culture medium (1 = original) were added to the adherent cells and the metabolic activity was measured after 24 h. A positive control (sodium dodecyl sulfate (SDS), Sigma) confirmed test sensitivity within the expected cytotoxicity range (0.05 to 0.4 mg/ml). Obtained activity values were related to the control (cell culture medium) and presented as percentage viability.

To assess cell adhesion and growth on hydrogels, 20,000 cells/cm^2^ were seeded onto the gel specimens. Metabolic activity was measured at 24 h and 72 h after seeding (CCK-8 assay). Population doubling time (t_D_) as measure for cell growth was calculated using the formula t_D_ = (t-t_0_) ln(2) / ln(A/A_0_) with t-t_0_ = 48 h, and A_0_ and A represent absorbance at 24 h and 72 h, respectively. For the fluorescent microscopy, samples (24 h and 72 h after seeding) were incubated for 5 min in propidium iodide (100 µg/ml) and fluorescein diacetate (6 µg/ml) in PBS, then observed using an Olympus CKX41 microscope equipped with a reflected fluorescence microscopy system (Olympus, Hamburg, Germany).

### Cell seeding

For the static culture, 3D-printed scaffold designs were optimised to support nutrition and oxygenation of cells inside the constructs. The investigation was conducted in static culture, as this culture model better reflects the tissue conditions at implantation site compared to dynamic conditions, especially in the initial phase after implantation, when perfusion by blood (bone phase) or joint fluid (cartilage or meniscal phase) is still not established or joint movement is restricted. The aim of these experiments was to evaluate the multi-material scaffolds as a regenerative construct by investigating key mechanism of tissue integration such as cell adhesion and distribution, proliferation and ECM formation for each material and design. Since the scaffolds were initially cell-free, cells were seeded on the surface of the scaffolds to emulate contact with the surrounding tissues (e.g., bone, cartilage) or cell-containing fluids (e.g., synovial fluid).

Gel-laden and gel-free scaffolds were loaded with chondrocytes by adding a 30 µl drop of cell suspension (16.6 × 10^6^ cells/mL) in a dry petri dish. To ensure uniform cell attachment, scaffolds were flipped every 15–25 min from all sides. After a maximum of 90 min, cell adhesion was complete, and the cell-seeded constructs were transferred to a multi-well plate and maintained in cell-specific cell culture medium. In the case of chondrocytes, the cell culture conditions were changed after 7 days from exclusively proliferative medium (RPMI-medium with FBS) to a chondrogenic medium (supplemented with 20 ng/mL TGF-β 3, PeproTech).

### Characterization of hybrid scaffold systems

#### Imaging techniques

The direct detection of adhered and proliferated cells was performed by epifluorescence microscopy. Seeded constructs were washed with PBS and stained with 3 µg/mL fluorescein diacetate (Merck, dissolved in PBS) for 15 min. For microscopy, an Olympus CKX41 microscope combined with a reflected fluorescence microscopy system was used (Olympus, Hamburg, Germany). The staining results were photo-documented using the ProgRes^®^ speed XT core5 camera and ProgRes^®^ CapturePro 2.10 software (both Jenoptik, Jena, Germany). At least 4 specimens per material type were tested.

Confocal laser scanning microscopy was performed using a DMI4000 inverted research microscope assembled to a TCS SPE-II confocal unit (Leica). The viability of attached cells was determined through live/dead staining using an ethidium bromide/fluorescein diacetate solution (10 mg/mL / 3 µg/mL, Sigma-Aldrich). After staining, scaffolds were placed upside down into a 4-compartment glass-bottom cell culture dish (Greiner Bio-One) and wetted with PBS. The closed dish was then mounted on the z-galvo micro-motorized microscope stage. Images were acquired using LAS AF v. 3.29702.1 software (Leica) with the following settings: 405 nm (20% intensity), 488 nm (20% intensity) and 561 nm (20% intensity) lasers, PMT and PMT Trans detectors, tile scan of 3 × 3 mm fields of view and 20 × 10 μm z-stack, 600 Hz scan speed, 10x magnification, ACS APO 10x/0.30 DRY objective, 94.3 μm pinhole, excitation beam splitter: QD 405/488/561/635. For presentation of viability and distribution of cells, image stacks were generated and a perspective representation was computed.

#### Biochemical assay

For the assessment of total glycosaminoglycan (GAG) and collagen content, one scaffold was digested with 1 mL of papain solution (Sigma-Aldrich, 0.125 mg/mL) containing 0.1 M disodium hydrogen phosphate, 0.01 M EDTA, and 0.01 M cysteine hydrochloride at 60 °C for 6 h. GAG quantity was measured spectrophotometrically using dimethylmethylene blue (DMMB) with chondroitin sulfate (Sigma-Aldrich) as a standard at 525 nm [25]. The hydroxyproline assay allows the direct measurement of collagen content [26]. Therefore, papain-digested samples were hydrolysed with hydrochloric acid (3 M, 24 h at 110 °C), oxidized with chloramine T reagent (0.056 M, 20 min at RT), and detected with Ehrlich’s reagent (1 M p-dimethylaminobenzaldehyde in isopropanol/perchloric acid (2:1 v/v), 15 min, 60 °C). Collagen quantity was measured spectrophotometrically at 595 nm with hydroxyproline (Sigma-Aldrich) as a standard. Collagen and GAG content were normalized to DNA amounts, measured spectrofluorometrically using the Quant-iT™ PicoGreen™ dsDNA Reagent (Invitrogen) as a standard. GAG and collagen contents were reported as µg/µg DNA.

#### Histological analysis

Scaffolds were embedded in carbowax (Sakura Finetek, Torrance, USA), snap-frozen in liquid nitrogen. and then cut into 10 μm slices. Since PDMS constructs did not adhere to glass or plastic slides, formalin-fixed constructs were dehydrated, embedded in paraffin and sectioned at 40 μm for free floating staining. To visualize cartilage- and meniscus-like GAGs, 0.7% safranin O (Sigma-Aldrich) in 66% ethanolic solution was used, and nuclei were counterstained with 0.2% Fast Green FCF (Sigma-Aldrich).

## Results

### Cytocompatibility of peptide hydrogels and 3D-Printed scaffolds

**Fig. 2:**
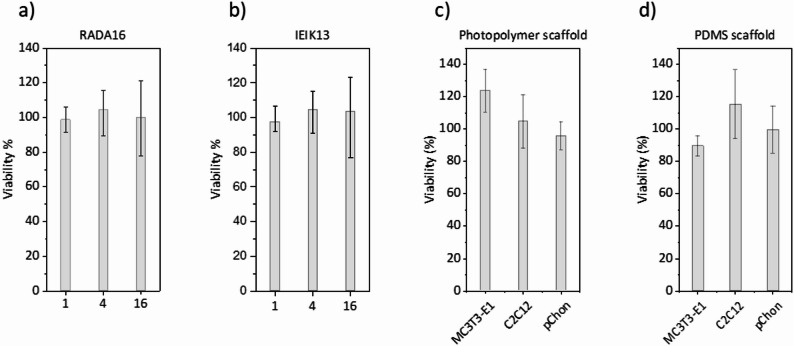
Cytotoxicity studies of the used scaffolding materials. **a**) - **b**) Diagrams showing the viability of MC3T3-E1 cells maintained with extracts and dilutions (1:4, and 1:16 in cell culture medium) of the peptide hydrogels. **c**) - **d**) Cytotoxicity test of 3D-printed scaffolds. Structures were extracted in cell culture medium for 24 h at 37 °C. Extracts were tested on MC3T3-E1, C2C12 murine cell lines and on porcine chondrocytes (pChon). Metabolic activity of the cells was measured after 24 h of treatment and related to the untreated condition (cell culture medium) given as percentage viability

Initial testing of the peptide hydrogels was conducted to optimize the cell seeding strategy. The solidification of the hydrogels is based on a change in ion concentration and the pH value. Preliminary studies showed that cells directly exposed to non-hardened gels, have implications on cell viability. Hence, the biocompatibility was assessed on gels under physiological conditions. For this purpose and to be comparable with the standard shape of the solid material, moulds in the shape of a cup with the same inner dimensions were used. After changing the ion concentration and pH value by incubating the gels in cell culture medium and changing the medium three times, extractions were initiated for 24 h. The obtained results did not reveal any cytotoxic effect. The viability of tested cells was comparable to those not exposed to extracts of both materials resulted in relative viability close to 100% (Fig. 2a, b).

The effectiveness of the sterilization protocols for the 3D-printed scaffolds was tested. Sterility test according to European pharmacopeia standard 2.6.1 did not show any microbial contamination after two weeks of maintaining the test structures in tryptic soy agar and thioglycolate broth test media, confirming sterility and effectiveness of the process. The cytotoxicity of the leachable components was tested using three different cell types (MC3T3-E1, C2C12, and porcine chondrocytes). The measurement of the metabolic activity (as a measure of cell viability) demonstrated that the fabricated 3D scaffolds were not cytotoxic, regardless of the cell type used, confirming their suitability as synthetic scaffolding structures for subsequent experiments focused on cartilage tissue engineering, as displayed in Fig. 2c, d.

**Fig. 3:**
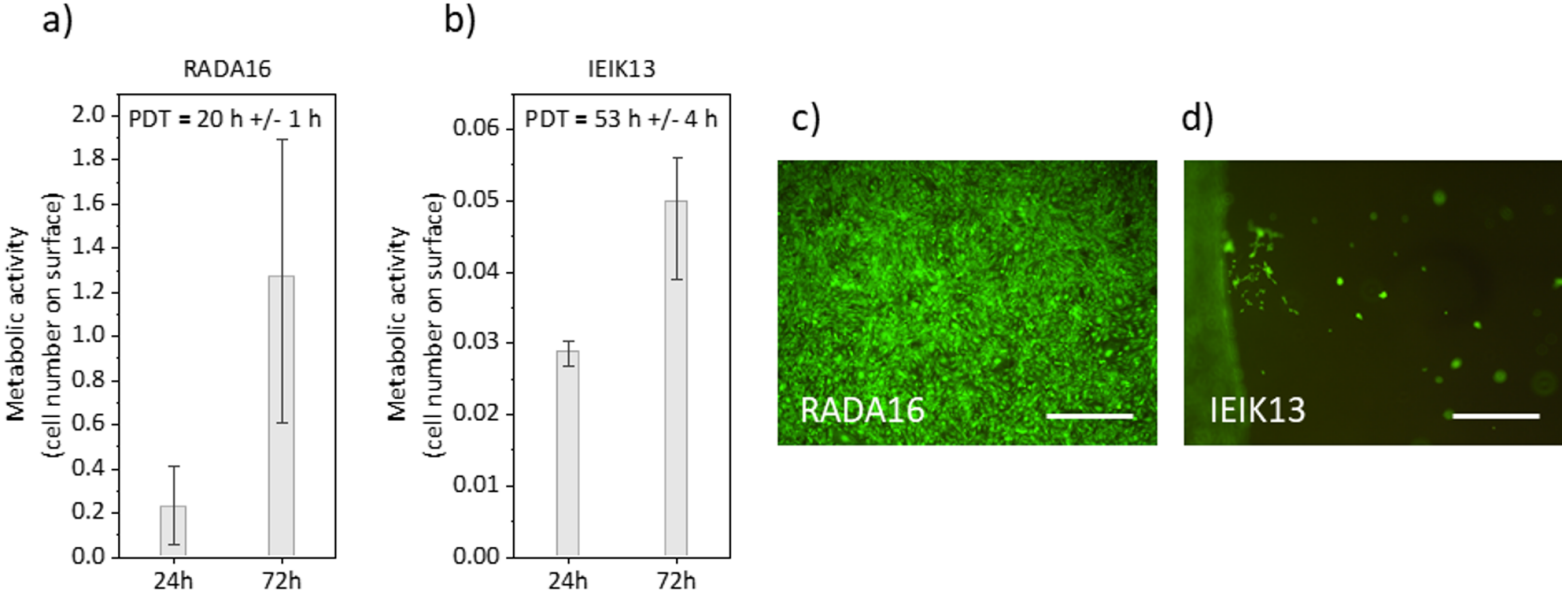
**a**) - **b**) Metabolic activity of adherent growing MC3T3-E1 cells seeded on plane RADA16 and IEIK13 hydrogel surfaces. Population doubling time (PDT) was calculated from metabolic activity shift. **c**) - **d**) Fluorescence microscopy images of viable (green) and dead (red) MC3T3-E1 cells grown for 72 h on the material surface. Scale bar = 500 μm

A clear difference between RADA16 and IEIK13 was observed when cells were seeded on the surface of the material. Whereas RADA16 was colonized with a high number of cells, only a small number of cells was detected on the surface of IEIK13 hydrogels indicated by metabolic activity measurements and fluorescence microscopy (Fig. 3). This observation indicates a lower cell-adhesive capacity of the IEIK13 hydrogel rather than reduced biocompatibility.

### Adhesion

**Fig. 4:**
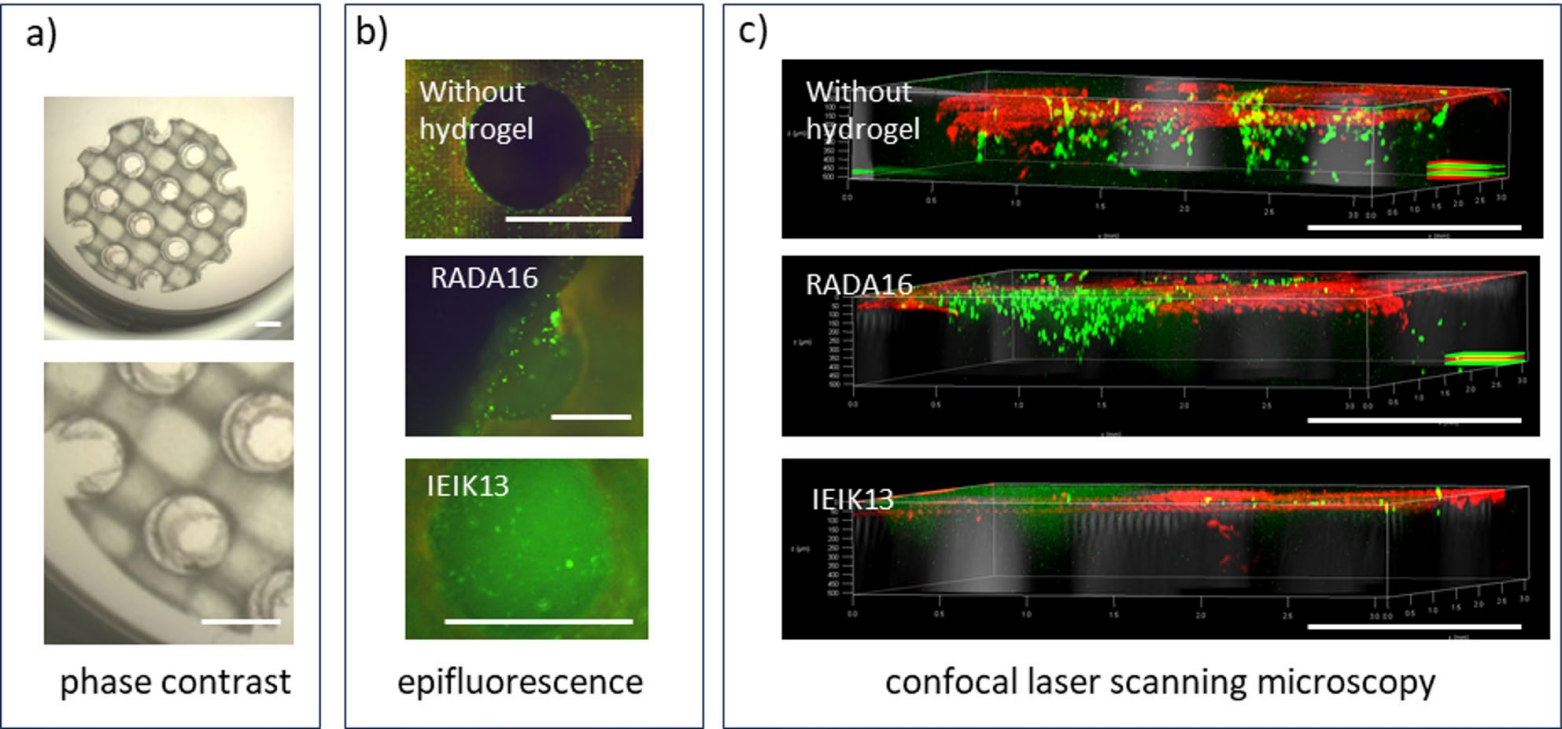
Microscopic analyses of photopolymer constructs after 21 days of maintenance in cell culture. (**a**) Gel-free, cell-laden porous test structures as an example of the maintenance of structural integrity of the constructs. Pores were still open and in shape as the whole construct. (**b**) Superficial inspection by fluorescence microscopy on gel-free and gel-laden constructs. Cells were stained with fluorescein diacetate and propidium iodide. Live cells appear as green dots, and dead cells as red dots. Green and red area stains arise from autofluorescence of the hydrogels and the photopolymer scaffolds. (**c**) Confocal microscopy demonstrated the presence of cells inside the pores up to a depth of 250–350 μm. In the gel-free specimens, the cells were found on the walls of the pores, whereas in gel-laden scaffolds, the cells were found in the center of the pores. Scale bar = 1.2 mm

**Fig. 5:**
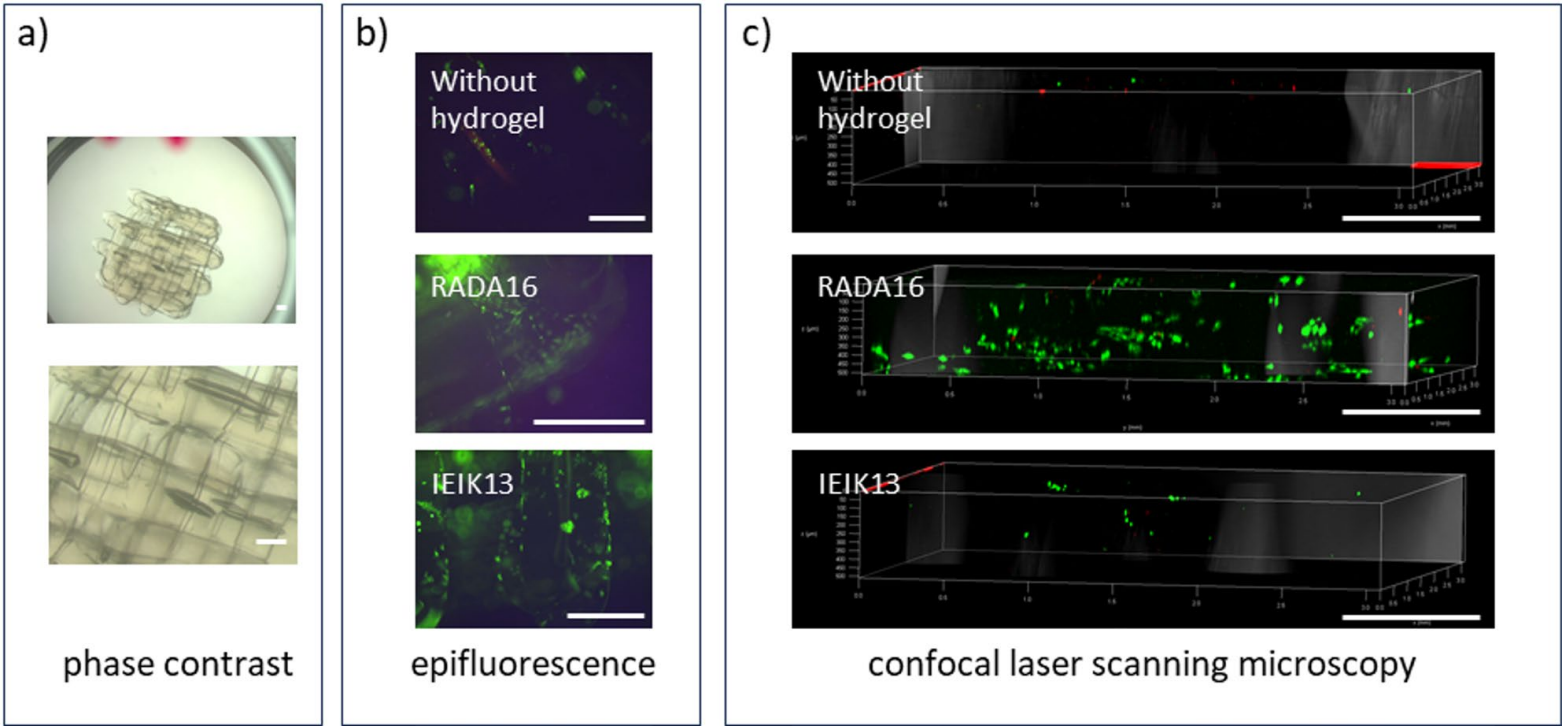
Microscopic analyses of PDMS constructs after 21 days of maintenance in cell culture. (**a**) Gel-free, cell-laden porous test structures as an example of the maintenance of structural integrity of the constructs. Pores were still open and in shape as the whole construct. (**b**) Superficial inspection by fluorescence microscopy on gel-free and gel-laden constructs. Cells were stained with fluorescein diacetate and propidium iodide. Live cells appear as green dots, and dead cells as red dots. Green and red area stains arise from autofluorescence of the hydrogels and the PDMS constructs. (**c**) Confocal microscopy demonstrated the presence of cells inside the pores up to a depth of 250–350 μm. In the gel-free and IEIK13-laden specimens, the cells were found predominantly on the surface, whereas in RADA16-laden scaffolds, the cells were found in the center of the construct. Scale bar = 0.8 mm

After 21 days of maintenance under cell culture conditions, the cell-laden scaffolds remained stable, and their structural integrity appeared unaffected. The pores were found to be open, and no part of the constructs was lost during media exchanges (Figs. 4a and 5a). Cells adhered to the photopolymer scaffold independently of the hydrogel. Superficial fluorescence microscopic inspection of live/dead-stained specimens demonstrated the presence of living cells adhering to the surface of the scaffolds (Fig. 4b). Confocal microscopy analysis of pore areas revealed the existence of living cells in deeper parts of the photopolymer scaffolds (Fig. 4c). While the cells on gel-free scaffolds were found on the walls of the pores as expected, cells in gel-laden structures were found in the center of the pores up to a depth of 250–350 μm, which is the detection limit of the technique, indicating that the gels allow cell migration. The fluorescence microscopy images showed that the photopolymer exhibited strong autofluorescence, appearing red. In confocal microscopy, this led to a false-negative representation of the living cells on the photopolymer surface. However, this effect was not observed in epifluorescence analysis.

The PDMS scaffolds showed excellent biocompatibility in long-term static culture testing but only enabled the adherence of chondrocytes to a limited extent. Epifluorescence imaging of live/dead-stained specimens demonstrated the presence of adhering cells on the surface of the constructs, but only to a significantly smaller extent in gel-free constructs compared to the gel-laden constructs (Fig. 5b). Confocal microscopy analysis of pore areas revealed the existence of cells in deeper parts of the constructs, with clear differences among the various conditions. While the cells on RADA16-laden constructs were found to be well distributed throughout the inner parts of the PDMS scaffold in high numbers, cells in gel-free and IEIK13 constructs were observed in only a few clusters. (Fig. 5c). The microscopic imaging results show that cells poorly adhered to the gel-free construct, and the hydrogel remarkably increased the number of cells detected both inside and outside the construct.

### Proliferation

**Fig. 6:**
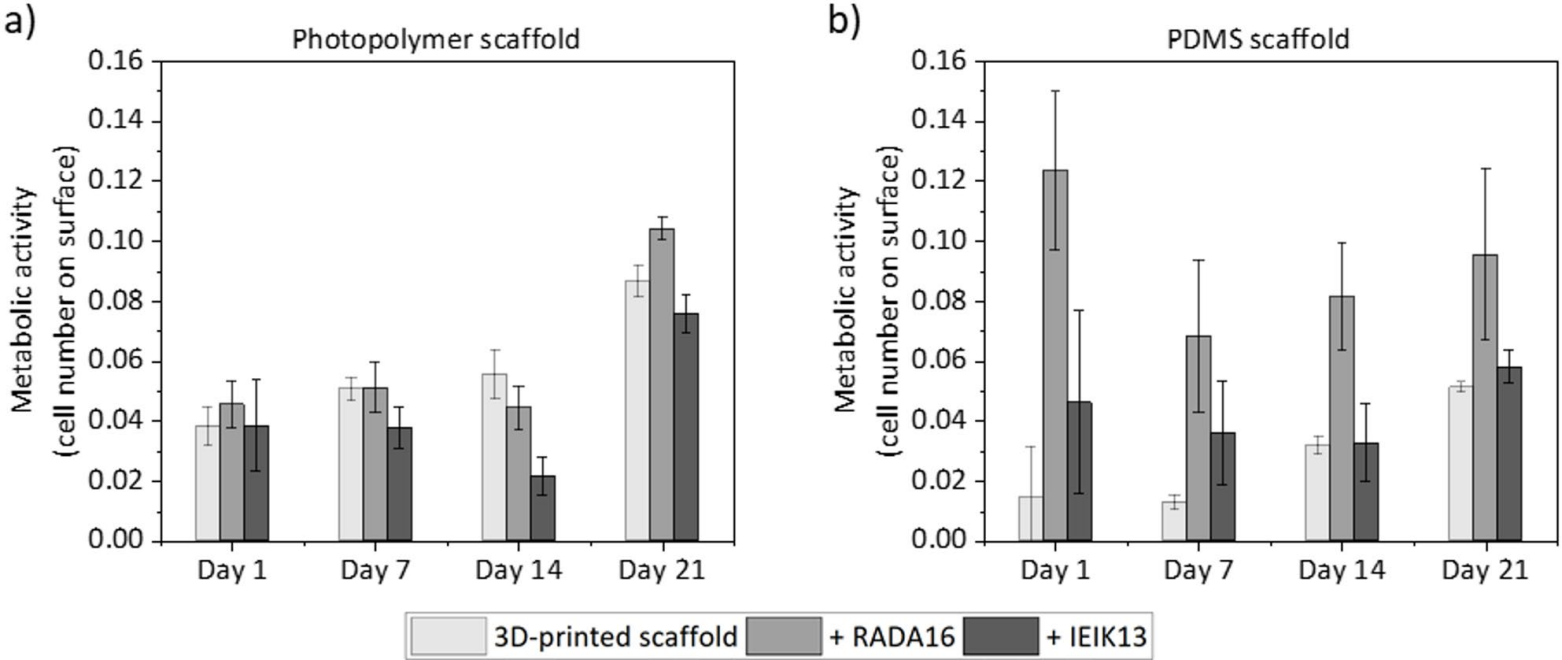
Cell proliferation on 3D-printed scaffolds and in hybrid configurations. Metabolic activity measured using the CCK8-assay at days 1, 7, 14, and 21 for (**a**) photopolymer scaffolds and (**b**) PDMS scaffolds augmented with hydrogels. All results were corrected by measurements of cell-free control cultures

The cell growth was monitored by metabolic activity measurements. The CCK-8 assay revealed that cell numbers in the gel-free photopolymer scaffolds continuously increased from day 1 to day 21, whereas cell numbers in hydrogel-laden scaffolds only clearly increased after 21 days (Fig. 6a). For the PDMS scaffolds, the hydrogels led to a high number of cells directly after seeding, on day 1. Overall, hydrogel-laden constructs showed an equal or higher number of cells at any measured time point compared to the gel-free condition (Fig. 6). Despite the large pore size of the 3D-printed scaffold, cells were able to proliferate within the hydrogel-filled pores. These findings suggest that while all groups supported cell proliferation over time, the presence of peptide hydrogels notably enhanced cell proliferation by day 21 compared to the other groups.

### Matrix formation

**Fig. 7:**
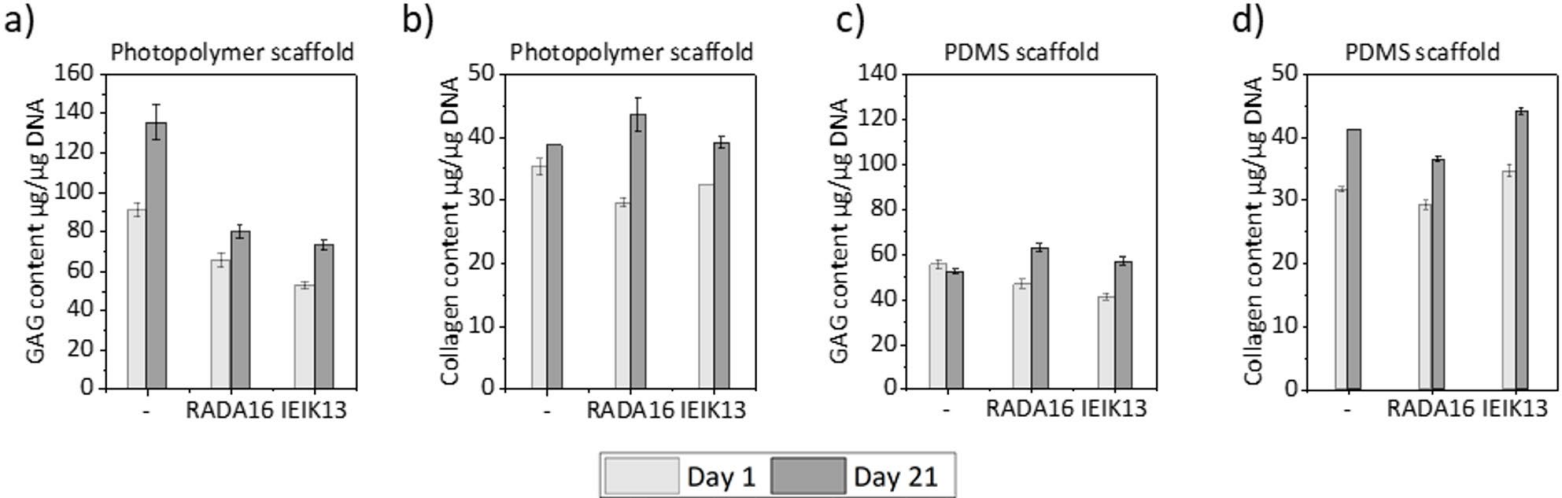
ECM formation on 3D-printed scaffolds and in hybrid configurations. (**a**) GAG content measured using the DMMB assay, in photopolymer scaffolds with and without hydrogel filling. (**b**) Collagen content determined via the hydroxyproline assay in photopolymer scaffolds with and without hydrogel filling. (**c**) GAG content in PDMS scaffolds with and without hydrogel filling. (**d**) Collagen content in PDMS scaffolds with and without hydrogel filling. All results were corrected by measurements of cell-free control cultures

Biochemical analysis revealed distinct trends in GAG content among the tested groups over the experimental period. The GAG and collagen content increased independently of gel loading for the photopolymer scaffold (Fig. 7a, b). Initially, the photopolymer scaffolds exhibited the highest GAG content at day 1, which continued to increase steadily until day 21 at an average rate of 2.22 µg GAG/µg DNA per day. In contrast, the 3D-printed scaffolds supplemented with peptide hydrogels also showed an increase in GAG deposition over time, though less prominently than the photopolymer, with an average rate of 0.71 µg GAG/µg DNA per day for RADA16 and 1 µg GAG/µg DNA per day for IEIK13. This trend suggests that while all groups supported GAG production, the presence of peptide hydrogels may have modulated the rate or extent of GAG accumulation within the scaffolds. Similarly, total collagen content analysis revealed a consistent upward trend across all experimental groups throughout the study period, reflecting ongoing ECM deposition and remodelling. The photopolymer scaffold demonstrated collagen accumulation from day 1 to day 21 with an average rate of 0.171 µg collagen/µg DNA per day. In contrast, the 3D-printed scaffolds supplemented with RADA16 and IEIK13 peptides exhibited a notably higher rate of collagen deposition, respectively 0.696 and 0.335 µg collagen/µg DNA per day, suggesting that these peptides enhance matrix formation over time. This underscores the role of peptide hydrogels in further promoting collagen synthesis and deposition.

Cells on PDMS scaffolds initially started to produce a cartilage-like ECM, which was detectable at the biochemical level (DMMB-assay for GAGs, hydroxyproline assay for collagens). The GAG formation was poor in gel-free constructs. In the case of RADA16, evidence of proteoglycan formation was observed at the histological level (Safranin O staining) (Fig. 9). The addition of the hydrogels RADA16 and IEIK13 proved to be superior to the hydrogel-free condition in terms of cell adhesion, proliferation, and ECM formation. The results confirm the findings from the microscopic assessment. The cell numbers were higher in constructs loaded with gels compared to the gel-free constructs, and RADA16 seemed to support cell adhesion and proliferation more effectively than IEIK13. Both hydrogels facilitated the formation of cartilage-like ECM (Fig. 7c, d).

### Colonization

**Fig. 8:**
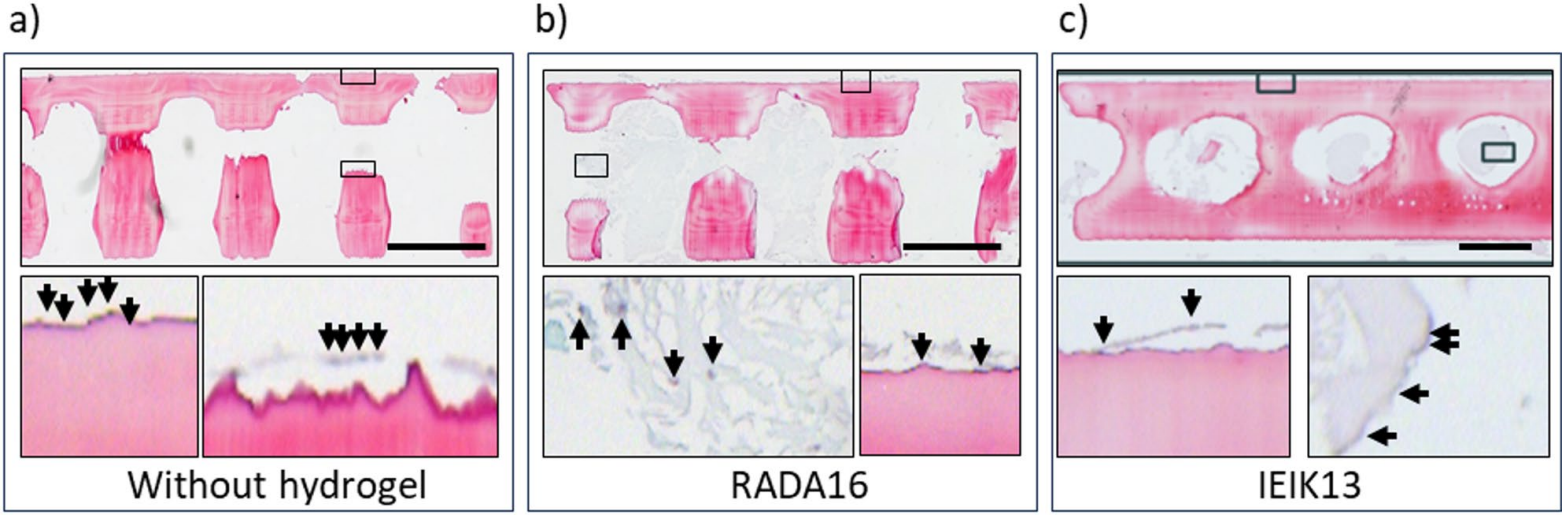
Safranin O / fast green staining of histologic sections of chondrocyte-seeded 3D-printed photopolymer scaffolds and in hybrid configuration at day 21. (**a**) Hydrogel-free scaffold with cells adherent to the inner and outer surface of the construct. (**b**) RADA16-loaded scaffold with green-stained gel on the surface and inside the scaffold. Cells were found on the surface of the photopolymer material as well as entrapped inside the gel in the inner part. (**c**) IEIK13-hydrogel was present inside the pores of the scaffold. Cells were found attached to the surface of the gels and the photopolymer scaffold. 10 μm section, arrows indicate cells. Scale bar = 1000 μm

Histological analysis of the constructs after 21 days of maintenance confirmed that chondrocytes extensively adhered to the inner and outer surfaces of the photopolymer scaffold (Fig. 8a). Cells were observed both within the hydrogel and adhered to the surface of the photopolymer scaffold. The RADA16 loaded constructs also enabled cell colonization in the central parts, as cells were found entrapped in the gels inside the pores (Fig. 8b). Within the hydrogel, cells appeared uniformly dispersed, maintaining their characteristic morphology. The hydrogel itself stained distinctively from the photopolymer scaffold, appearing as a lighter, less dense region colonized by cells. On the surface of the photopolymer scaffold, cells adhered and spread, demonstrating interaction with the scaffold material. This dual localization of cells within the hydrogel and on the photopolymer highlights the scaffold’s capability to support cell growth.

**Fig. 9:**
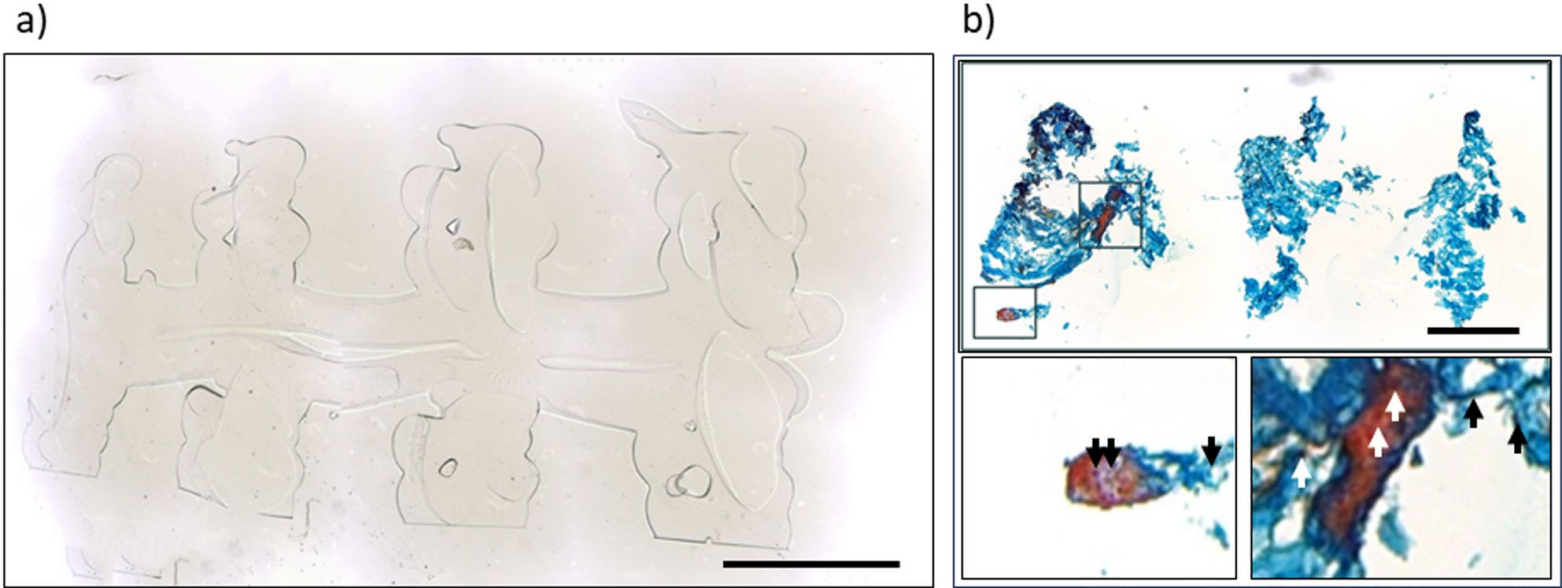
Safranin O / fast green staining of histologic sections of gel-laden chondrocyte-seeded 3D-printed PDMS scaffolds and in hybrid configuration at day 21. (**a**) A stained 40 μm section of RADA16-laden scaffolds prepared as free-floating staining. Despite fixation, the hydrogels were lost, and only the PDMS scaffold was found. (**b**) Staining on slides led to the loss of the PDMS during staining, despite the application of different fixation agents, but the hydrogel attached and could be stained. The gel fragment contained cells, and in cell-rich areas, a red staining was observed, indicating the presence of proteoglycans. These areas are located in the central and superficial regions of the construct. Arrows indicate cells. Scale bar = 1000 μm

Histological analysis of the PDMS constructs proved to be difficult, since PDMS did not adhere to any carrier material and the hydrogels were lost during free-floating staining, making the combined analysis of hydrogel and PDMS impossible. The Safranin O staining for proteoglycans of the PDMS did not reveal any meaningful information, since cells and possible ECM seemed to be lost with the detachment of the hydrogel (Fig. 9a). For RADA16, the presence of cells and proteoglycan was demonstrated in the surface and central regions of the constructs (Fig. 9b). In the case of IEIK13, the preparation of tissue sections was not possible, since the hydrogel was lost in all cases. However, biochemical analysis of GAGs and collagen (Fig. 7c, d) and metabolic analysis (Fig. 6b) indicated that cells were present and ECM had formed.

## Discussion

The 3D-printed synthetic scaffolds in this study featured a pore size ranging from 0.8 to 1.2 mm, which was selected based on the technical limitations of the printing materials and fabrication technologies. Such large pores can hinder effective cell attachment and matrix formation, as they provide insufficient mechanical support for chondrocytes. To address these limitations, the large pores were manually filled with peptide hydrogels, namely RADA16 and IEIK13, composed of self-assembling peptide amphiphiles. The peptide hydrogels were incorporated into 3D-printed scaffolds by immersion, allowing the initially viscous, self-assembling hydrogels to solidify upon exposure to physiological pH and ionic conditions, resulting in physical entrapment around and within the scaffold pores. This approach facilitates the rapid creation of hybrid constructs and forms a nanofiber network both around and within the scaffold’s pores, creating biomimetic environments that closely resemble natural tissue structures favourable to harbour cells and provide physiological environment. The specific biological properties of each hydrogel can be used to influence the colonization and cell distribution of a 3D-printed scaffold. While both hydrogels are suitable to enhance bioactivity of 3D-printed scaffolds, RADA16 demonstrated superior support for cell adhesion, proliferation, and migration compared to IEIK13 in the chosen model. Further investigation into the structural differences between RADA16 and IEIK13 could provide valuable insights into tailoring hydrogels to optimize specific cell functions. This difference can be attributed to their distinct amino acid composition and resulting nanofiber architecture. RADA16 and IEIK peptides both self-assemble into β-sheet–rich nanofibrous hydrogels through a combination of hydrophobic and electrostatic interactions, but their supramolecular properties differ due to their amino acid compositions. RADA16, built on alternating arginine, alanine, and aspartic acid residues, forms relatively soft and hydrated networks because alanine is only mildly hydrophobic and contributes to flexible fiber packing. In contrast, IEIK peptides incorporate isoleucine, a much more hydrophobic and bulkier residue, which promotes tighter hydrophobic collapse and more densely packed β-sheets. Together with strong E–K electrostatic pairing, IEIK peptides typically generate denser, more rigid, and mechanically stronger fibers than RADA16. As a result, IEIK hydrogels generally exhibit higher stiffness and structural stability compared to the more compliant RADA16 matrices.

The impact of the peptide hydrogel on cell colonization is particularly evident when comparing the inert PDMS scaffolds with and without hydrogel loading. In the absence of hydrogel, PDMS supported only sparse surface attachment, whereas RADA16-laden constructs showed a dense and homogeneous cell presence throughout the pores. This qualitative observation from fluorescence and confocal microscopy (Fig. 5b, c) was corroborated by the biochemical data, where glycosaminoglycan and collagen contents markedly increased only in hydrogel-augmented PDMS scaffolds (Fig. 7c, d). These findings demonstrate that the peptide hydrogels substantially enhance cell colonization, a result made especially clear by the use of the otherwise non-adhesive PDMS matrix as a functional control.

One significant advantage of our method is its scalability and ease of implementation. The 3D printing processes for the synthetic scaffolds, DLP and liquid deposition modeling, are both rapid and accessible. This is especially relevant for osteochondral and meniscal defects, which often require customization to meet the specific needs of individual patients [27–29]. The subsequent hydrogel filling process is straightforward. The sequential loading process offers distinct advantages, such as sterilizing individual components separately, followed by their assembly in a sterile environment. Fabricating the 3D-printed scaffold separately may facilitate meeting regulatory standards and quality assurance criteria. Furthermore, since printing does not need to occur under sterile conditions, this reduces complexity and enables refinement of scaffold designs in non-sterile settings. Alternative technologies, such as two-photon polymerization (2PP) or material jetting, offer unique advantages but come with notable limitations. Although 2PP can achieve higher resolution and smaller pore sizes, it is considerably slower, rendering it less practical for large-scale applications [30]. Material jetting, while also a potential option, presents challenges such as the risk of unintended mixing when printing multiple materials together [31]. Additionally, the lack of fully biocompatible support materials can lead to cytotoxicity issues, especially if residual support material becomes trapped within the porous scaffold. These factors make DLP and extrusion printing the preferred choices for producing scaffolds that are both functional and biocompatible for cartilage tissue engineering. In addition, the workflow allows, that all components can be sterilized easily, and the absence of pre-seeded cells or growth factors reduces the complexity of scaffold preparation, aligning with practical and regulatory considerations.

PDMS’s role in biomedical applications can be significant when used strategically [30] especially for cartilage tissue engineering [32, 33]. Traditionally, PDMS is not considered suitable for tissue engineering due to its inherent lack of cell affinity. However, through augmentation with a hydrogel, PDMS scaffolds can provide an environment for cells within their pores and improve cell colonization.

Finally, the proceedings aimed to test whether cartilage-like ECM can be formed within the scaffolds. The applied in vitro model (seeding cells on material surface) was deliberately kept simple to accomplish studying a large number of samples. Furthermore, the static cell culture model enabled the analysis of a multitude of material specimens in a more reproducible and comparable fashion. The aim of the static cell culture experiments was to evaluate the readiness of the developed manufacturing pathways to act as a regenerative scaffold by investigating basic mechanism of tissue integration such as cell adhesion and distribution, proliferation and ECM formation. As porcine chondrocytes tend to lose the chondrogenic phenotype by extensive proliferation in vitro (similar to how human MSCs also lose the chondrogenic capacity), after one week of maintenance following seeding on materials, TGF-β 3 was added to the medium to induce a return to a more chondrogenic phenotype. While biochemical and histological analyses (GAG, collagen, and Safranin O staining) indicate the formation of a cartilage-like ECM within the constructs, these data do not directly confirm chondrogenic differentiation at the molecular level. The presence of TGF-β3 in the culture medium and the observed accumulation of sulfated glycosaminoglycans and collagen suggest the maintenance or partial restoration of the chondrocytic phenotype under static conditions. However, future studies should include immunofluorescent or immunohistochemical staining for cartilage-specific markers such as collagen type II and aggrecan, as well as gene expression analyses (COL2A1, ACAN, SOX9), to substantiate the chondrogenic potential of the peptide hydrogels within the hybrid scaffolds.

The histological results verified that the hydrogels within the 3D-printed scaffolds remained stable, effectively entrapping cells for up to 21 days without premature degradation. This stability under in vitro conditions simulates physiological environments, providing insight into the hydrogel’s durability and performance in the presence of relevant cell types.

Although the present study was conducted under static culture conditions, dynamic loading is known to influence chondrocyte behavior and hydrogel–scaffold interactions. Cyclic compression or shear can enhance nutrient diffusion and promote a more uniform cell and matrix distribution within hybrid constructs. In future dynamic culture models, such mechanical stimulation could further reinforce the integration between the 3D-printed scaffold and the peptide hydrogel. Regarding material stability, both RADA16 and IEIK13 consist of natural amino acids and are gradually degraded by enzymatic activity under physiological conditions. RADA16 typically exhibits a slower degradation rate due to its dense β-sheet nanofiber network, whereas IEIK13 forms softer, less densely packed fibrils that are expected to degrade more rapidly. This controlled biodegradation is advantageous for cartilage regeneration, as it allows progressive replacement of the hydrogel by newly synthesized extracellular matrix during tissue remodeling.

However, the study focused on modelling the early time points of cartilage formation. Even without mechanical loading, we were able to observe that cells migrated into the scaffolds, indicating some degree of cellular response to the static environment. Future studies should incorporate dynamic culture systems or bioreactors to evaluate the hybrid scaffold system’s performance under mechanical stress. Additionally, long-term studies examining in vivo degradation profiles and ECM formation will be necessary to assess the clinical viability of these constructs.

## Conclusion

This study highlights the potential of rapid 3D printing techniques, in combination with self-assembling peptide hydrogels, to enhance cartilage repair strategies. Adopting fast 3D printing makes scaffold fabrication practical and accessible, while the integration of peptide hydrogels effectively overcomes bioactivity limitations, supporting both cell adhesion and ECM formation. Comparative analysis shows RADA16 offers stronger support for cartilage-like ECM formation, while both RADA16 and IEIK13 notably improve PDMS biocompatibility, supporting cartilage-like ECM development and promising advancements in tissue engineering.

## Data Availability

The datasets used and/or analysed during the current study are available from the corresponding author on reasonable request.

## Abbreviations

PCL: Poly(ε-caprolactone)
PLA: Poly(lactic acid)
PLGA: Poly(lactic-co-glycolic acid)
PDT: Population Doubling Time
BMP: Bone Morphogenetic Protein
ECM: Extracellular Matrix
DLP: Digital Light Processing
PEG: Polyethylene
PDMS: Polydimethylsiloxane
EtOH: Ethanol
FBS: Fetal Bovine Serum
RPMI: Roswell Park Memorial Institute
EDTA: Ethylenediaminetetraacetic Acid
CCK-8: Cell Counting Kit-8
PBS: Phosphate-Buffered Saline
SDS: Sodium Dodecyl Sulfate
TGF: Transforming Growth Factor
GAG: Glycosaminoglycan
DMMB: Dimethylmethylene Blue
pChon: Porcine Chondrocytes
2PP: Two-Photon Polymerization
